# The Dublin Hospital Reports, and Communications in Medicine and Surgery. Vol. Fourth

**Published:** 1827-07

**Authors:** 


*The Dublin Hospital Reports and Communications in Medicine and Surgery.* Volume the Fourth. 8vo. pp. 600, with numerous plates. Dublin, London, Edinburgh, May, 1827.

The progress of this excellent work appears to have been interrupted, or at least retarded by the death of one of its editors—the late Professor Todd, and by the removal of another, the late Dr. Edward Percival, from Dublin to Bath. The establishment also of a contemporary pub-

---

* We are not one of those that would, on all occasions, spur a generous horse to a leap which he cannot accomplish, hence we have overlooked, as we usually do, all insignificant inaccuracies in the above work, judging of it favourably as a whole and not in part, but anxious for precision of language, important as the real index of a writer's mind, and still more important as influencing the minds of readers, we are compelled to observe that Dr. Baron, while writing on vaccination, expresses himself with singular looseness, and seeming incorrectness, by frequently using the term *pustule*. For instance,—" very different from the benign solitary *pustule* which characterizes the variolæ vaccinæ."—p. 244.—Now we had always understood that the genuine cow-pox was a vesicular disease, exhibiting lymph; and not pustular, containing pus. To prove we were not very wrong, and had at least high authority to support us, we shall extract strong proofs from an author, among numbers, entertaining similar opinions.—" The characteristic of this (vaccinia) is a semi-transparent, pearl-coloured *vesicle* :"—" is filled with *clear lymph.*"—" The *pustule,* which is sometimes produced *instead of the proper vaccine vesicle,* is more like a common festering boil."—Again,—" Three varieties of irregularity (in vaccination) have been noticed; namely, *pustules,* ulcerations, and vesicles of an irregular form."—*Bateman's Synopsis of Cutaneous Diseases, 2d Edition, pp.* 213, *et seq.* 1813.—We hope Dr. Baron will attend to this subject, and either correct his own error, or that of others, since error there must be somewhere.—*Rev.*

lication (Transactions of an Association, &c.) must have operated in preventing many communications passing through the present channel, and thus in delaying the completion of its volumes. Still, with all these difficulties and disasters, the DUBLIN HOSPITAL REPORTS have not succumbed, and the present volume is not inferior—indeed, we think it superior to most of its predecessors. From the initials at the close of the preface to the present volume we must suppose that Dr. Cheyne and Dr. Colles are the editors :—And although they have not contributed very largely, in their nominal characters, to this portion of the series, yet they deserve the thanks of the profession for bringing forward such a mass of rich materials as the present volume contains. We could not procure a copy of the work in time for an extended analysis in this number; and we have to complain of a certain degree of *favouritism* practised on the present occasion, by "*a person or persons unknown*," whereby the work was *reviewed* by a northern contemporary long before it was regularly *published*. This is a manœuvre unworthy of any concerned in so respectable a publication—and it will probably not be repeated. We speak not on our own account—for our object has never been to get the *first* notice of a new volume out in our Journal—but to get the *best* account—that is, an account the most minutely analytical, yet divested of all unnecessary verbiage. Such an object is, at all times, incompatible with rapid and early reviews of works; but of far more importance to the public and to ourselves.

In our analyses of this volume we shall not follow the order in which the articles are arranged in it; although none of them shall be passed unnoticed in the sequel.

### ART. I.

*Clinical Observations.* By ROBERT JAMES GRAVES, M.D. Fellow of the College of Physicians, &c. &c. &c.

1. *Abscess of the Liver.* A robust man was admitted into the Meath Hospital, with well marked symptoms of acute hepatitis, which resisted the most vigorous antiphlogistic means, and, for four weeks after the subsidence of the first attack, the symptoms indicated unequivocally the formation of an abscess in the organ. Hectic fever, with rigors, night sweats, and emaciation, were attended by constant sense of uneasiness and weight in the hypochondrium, which was fuller and harder than natural. The tenderness and pain, at first more diffused, became after a time, confined almost to one spot, nearly corresponding with the centre of the external elevation. Poultices were applied; but the abscess shewed no disposition to point; the swelling remaining stationary, and the integuments preserving their natural colour. The constitution was giving way, and the question of an operation was necessarily agitated. It was resolved to try the effect of an incision carried some way through the integuments, but not so deep as the matter, if that should be found to lie at a great distance from the surface. An incision about four inches long was made by Mr. Mac Namara, exactly over the centre of the tumour. The abdominal muscles were found of considerable thickness, and quite healthy; and although the incision was carried very deep, yet the situation of the hepatic abscess was not felt more distinctly; "so that it now became quite evident that no prudent surgeon would have persevered in an attempt to open directly into it." In two days afterwards, a burst of matter came forth, from a fit of sneezing, not immediately from the bottom, but from one side of the wound. Pressure on the liver caused the matter to flow abundantly; and this continued more or less for several weeks, the man recovering perfectly in the end.

The above procedure is considered not only to be novel, but also to be an improvement on the practice of opening deep-seated abscesses, especially of the liver; and its adoption is recommended by Dr. G. to our Indian practitioners, who have frequent opportunities of testing its utility.*

2. *Rheumatism of the Temporal Muscles.* Our author has seen two cases, in which the rheumatism seemed to be confined almost entirely to the temporal muscles, the consequence of which was, an elevation of the lower jaw, the latter being firmly pressed against the upper jaw, so that no food, except in a fluid state, could be introduced. This affection was unattended by any constitutional derangement, but caused much uneasiness in the minds both of the patients and their friends, being by them confounded with locked-jaw. The cure of this complaint is easily effected by the application of leeches to the region of the temporal and masseter muscles, and by the internal use of anti-rheumatic remedies.

The above disease is not exceedingly rare. We have seen three or four instances of it, and one very recently in the person of a lady who was under the care of Mr. Guthrie and Dr. Johnson for another and a much more serious malady. The lady underwent a surgical operation, which rendered the locking of the jaw more suspicious; but she was quite unapprehensive, as this phenomenon occurred whenever she had rheumatism about the head. It went off in a few days, without any remedy, except a stimulating liniment to the surface.

3. *Idiopathic Glossitis.* A medical student applied to Dr. Graves, who found him labouring under severe febrile symptoms of a week's continuance, attended with great pain in the neck and occiput, relieved by epistaxis. The left half of the tongue became very tender and painful, with gradual increase of size. When Dr. G. first saw the patient, it was enormously swollen, and nearly filled the mouth, which could scarcely be closed on account of the protrusion of the tongue. The *right* half of the organ was perfectly natural, and its comparative diminution formed a striking contrast with the size of the *left* side. Two or three applications of six leeches at a time to the inflamed half

* See an interesting case of hepatic abscess bursting into the chest, and cured by paracentesis thoracis, in another part of this Number.

(part of which appeared to be approaching to gangrene) produced a speedy cure, though there is still some tumescence of that side of the organ, after a lapse of two years.

Idiopathic glossitis is very rare; a few cases only being recorded of late years on the Continent, and in none of these was the inflammation confined to one half of the tongue. Neither does it appear to have occurred to the medical attendants to apply leeches to the tongue—a mode of depletion preferable, we think with Dr. Graves, to any other.

4. *Colica Pictonum.* Two cases of this disease, in a very violent form, were treated by Dr. Graves at the Meath Hospital, and speedily cured by strong tobacco fomentations, applied to the abdomen till the peculiar effects of this herb were manifested in the system—and cathartic pills, containing oil of croton, assisted by injections. By these means, copious discharges were obtained from the bowels, with remission of the pain. In a case of paralysis succeeding colica pictonum, much benefit was derived from the use of strychnine, as recommended by Magendie.

5. *Whitish Stools.* This disease is what would have formerly been called "*fluxus cœliacus*" and the colour attributed to the admixture of chyle in the motions. Nobody, now-a-days, dreams of explaining the pathology of the disease on such a basis, though the late learned Dr. Good still retained this mode of explanation amidst a vast deal of other lumber in his Study of Medicine. But to the case in question. A gentleman applied to our author, labouring under the following symptoms, having previously experienced a severe attack of dysentery, viz.—good appetite with apparently good digestion, yet with progressive emaciation and loss of strength—one or two natural motions daily, and ten or twelve other calls to stool, attended with irresistible bearing down and weight about the rectum, the evacuation often taking place before he had time to get to the water-closet. These evacuations consisted merely of two or three spoonfuls of muco-gelatinous matters, generally resembling thick milk or puriform matter, and occasionally a transparent jelly. "This fluid was evidently a secretion from the mucous membrane of the rectum in a state of irritation or sub-inflammation:"—a condition, Dr. G. observes, of the mucous membrane, which constitutes the disease denominated "*chronic blennorrhagia,*" in the urethra, and the "*fluxus cœliacus,*" when seated in the rectum. This is supposed to be the disease described by Dr. Baillie, and alluded to at page 155 of this Number. But we cannot admit this identity of the two diseases, at least on the pathology maintained by Dr. Graves, because, in the article alluded to, we have shewn that, in two cases, dissection detected no disease in the mucous membrane of the colon or rectum. Besides, the disease described by Dr. Baillie is not generally attended with much emaciation, and "the alvine discharges resemble a mixture of lime and water," which is a species of discharge very different from that described in the present case.

Be this as it may, our author tried various remedies without success, until, at length, he resolved to exhibit strychnine, on the authority of Dr. Rummel, who had employed the extract of nux vomica with great advantage in this complaint. One twelfth of a grain of strychnine was given twice a day, by which the cure was effected in about three weeks. If this be a case of that dangerous malady described by Dr. Baillie, we have here another remedy added to the one mentioned in the Periscope of this Number, as tested by Dr. Elliotson at St. Thomas's Hospital.

6. *Black, or very Dark Stools.* These may be caused, first, by an effusion of blood into the intestines, causing true melæna:*—Secondly, by black bile. Formerly the *atra bilis* was looked upon as the sole cause of black stools; but Hoffman and Home demonstrated that they were sometimes caused by blood. But, in fact, the number and variety of dark and disordered secretions from the mucous membrane of the stomach, bowels, and liver, are almost interminable.

In a case which came under our author's care, and in which very large quantities of matter, sometimes like tar, and sometimes resembling ink, were passed by stool, for ten or twelve days in succession, "the black colour was evidently derived from the mucous membrane." A frequent examination of the discharges shewed that this colour was not derived from the blood; "for it was quite evident that, in such a case, the blood could not have remained in the intestines very long after its effusion, for the stools were frequent and copious—and I know by experience, that, in true melæna, blood which has been retained, even for a considerable time, in the intestines, will tinge water red, which was not the case here." In this case, too, there was not that debility and frequent fainting which follow melænic evacuations, if very copious. On the contrary, the discharge of these black matters gave relief in this case, as it generally does in such cases.

The disease finally yielded to oil of turpentine and stimulating tonic medicines; but was not ameliorated by mercurial preparations. The following experiment proves, Dr. G. thinks, that the black matter passed by stool, was a vitiated secretion from the mucous membrane of the intestines, and neither of a melænic or biliary nature.

"I cleaned one half of the tongue, from which I washed, with much difficulty, the black tenacious mucus. I watched it for several hours, and found that the part I had cleaned became gradually black and foul, the black mucus being evidently a secretion from its surface.

---

*Note, by the Editors of the Transactions.*

* "The blood effused in melæna coagulates in the bowels, and, being exposed to heat and air, turns black, and often becomes fetid. When retained very long, the colouring matter may be washed away, and the coagulated fibrin left. In a dissection of a woman who died of melæna, at Berlin, we found, in the large intestines, many hard balls, the size of apples, and consisting of fibrin, deposited in concentric layers, evidently the result of successive separations from the blood, effused during several different attacks."

Analogous to this case, is one which was formerly under the care of Mr. Wilmot, and in which large quantities of blackish mucus were discharged *from the bladder*." 53.

7. *Contagious Psoriasis.* It is stated by Bateman and others, that scaly diseases are not contagious. Our author lately met with a case which appears to him to prove the contrary, under certain circumstances. A gentleman had been affected with psoriasis palmaria for some years previously to his residing in Dublin. In the course of the last year, Dr. G. was called to see this gentleman's butler, who had contracted an extensive psoriasis on the back of his hand, and which the butler attributed to his wearing his master's old gloves. In a short time afterwards, the house-maid became affected with the same complaint, which she attributed to contact with her master's linen, &c.

The most extensive case of psoriasis diffusa which Dr. Graves ever saw, occurred in a boy, after sleeping on some fleeces of wool without his shirt. In this case, he doubts whether the disease was produced by the irritating qualities of the wool, or some disease in the sheep, from whose backs the fleeces of wool were taken. He has seen an entire family of children infected with a disease resembling the itch, from playing with a mangy dog.

It is hardly necessary to observe that, although the probability is in favour of the psoriasis, in the former case, being derived from the master to the servants; yet there is nothing like certainty in the case, as it rests a good deal on the *post hoc ergo propter hoc* argument. We are not among those, however, who deny that psoriasis, or, indeed, almost any cutaneous disease, may occasionally assume a contagious character.

8. *Swelling of the Extremities.* Besides phlegmasia dolens and elephantiasis, (bucnemia tropica, or Barbadoes leg) our author has observed two sorts of tumefaction, " which are pathologically different from either of the above species, and occasionally produce a monstrous degree of deformity."

*First Species.* Dr. G. has only seen one instance of this, which appears to be similar to two cases published, one by Mr. Chevalier, in the 2d volume of the Medico-Chirurgical Transactions, and one by the younger Söemmering. In Mr. Chevalier's case, the origin of the disease seemed connected with a previous attack of phlegmasia dolens. Dr. Graves's patient was a young man, 25 years of age, admitted into the Meath Hospital, on account of an extraordinary enlargement of the left lower extremity. The limb measured (below the knee) two feet, nine inches, at its largest part—in fact, it was there about the size of his own body. The swelling felt firm to the touch, and extended nearly half way up the thigh, when it suddenly diminished, and there the integuments felt loose and flabby. The upper part of the thigh was not increased in size. On the foot, the swelling was similar to that described by Mr. Chevalier, and overhung the toes. Some parts of the skin were covered with hard concretions, arising from an occasional oozing of fluid from slight fissures, drying and forming scabs, which mingled with the furfuraceous scales of the desquamating cuticle. Towards the bottom of the deep fissures which divided the swelling into lobes, the cuticle was very thin, and the skin reddish, and always moist. The swelling had commenced several years previously, and had gradually attained this enormous size, unattended by pain or inflammation of skin, adipose tissue, or inguinal glands. The knee and ankle joints were flexible and he could walk, run, and (till an excoriation on the back of the leg prevented him) gain his bread by labour. His virility was unimpaired. From the perfect action of the muscles in this and in Mr. Chevalier's case, it is probable that the disease had not invaded the interstices between the muscles. Dr. G. has no doubt that the tumefaction arose from an extraordinary growth of the skin and subjacent adipose tissue. He, therefore, considers the disease as totally different from phlegmasia dolens and the Barbadoes leg, "both of which arise from inflammation." A good plate of this disease is given.

*Second Species.* This kind of enlargement, which is by no means uncommon in Ireland, may affect either upper or lower extremities, and attains such a size as to render the limb useless. It has not been observed, except among the poor—but among them very frequently. The first appearance of swelling is generally preceded by febrile symptoms and well-marked gastric derangement, in a day or two after which, the back of the hand and wrist become swollen, and hotter than natural—the skin red in slightly elevated patches, which redness disappears on pressure. Vesications never form, and he has known but one instance of suppuration taking place. In a few days, the heat and redness subside, and the patient keeps his usual health, the slight œdema soon vanishing; but repeated attacks leave a permanent œdema. In a year or two, the hand is swollen to two or three times its natural bulk, the increase of bulk being principally on the back of the hand. Finally, the skin becomes thickened and corrugated, particularly about the wrists; and, in the advanced stages of the disease, rhagades and painful fissures are occasionally formed during exasperated attacks. Much fluid oozes out and scabs are formed. Dr. Graves labours to prove that this disease and the first species are different from true elephantiasis; and we think him correct in making this distinction.

Although the accompanying fever is often attended with gastric symptoms, yet there are other cases in which no symptom of stomach disorder exists. The fever always appears before the local affection—"the former, therefore, is probably the cause of the latter." Dr. G. shrewdly suspects that, from the intermitting nature of the fever—its greater frequency in some places than in others—and its prevalence in Spring and Autumn, the febrile complaint may partake of the *aguish* character.

In respect to treatment, when the case is not of long standing, considerable advantage is derived from the antiphlogistic plan,—purgatives—leeches—cold lotions—saturnine washes, &c. During the intermission, we must employ rest, bandages, bark, and, if that fail, arsenic. The antiphlogistic plan should be resumed the moment that inflammatory symptoms are evinced.

As we shall have occasion to introduce Dr. Graves again, on several occasions, in the course of our review of the present volume, we shall only say that the profession are under many obligations to this zealous and talented physician for the facts which he has so accurately observed and faithfully recorded in the work now before us.

### ART. II.

*Case of remarkable Pulsation in the Veins.* By CHARLES DAVIS, M.D. Surgeon to the North-East Dispensary.

The patient in whom this phenomenon appeared, was a young girl, six years of age, whom Dr. D. found with symptoms of acute hydrocephalus. She was much emaciated—had had hooping-cough four months previously, from which she never perfectly recovered—and, for the last ten days, was affected with bilious vomitings. She was now listless—tongue coated—complete loss of appetite—dull pain in the forehead—pupils somewhat dilated, but contractile on the application of light—skin hot and dry—pulse irregular and 88, full and strong—bowels constipated—stools green and hydrocephalic—an eruption, resembling purpura simplex, scattered over the skin, and a vesicular eruption on the chest and neck. In all the veins, there was a distinct and well-marked pulsation, synchronous with that of the arteries, and, in the veins of the extremities, visible to the naked eye. The veins were rather larger than usual, and pressure on any of them stopped, of course, the pulsation above the point pressed, and increased it below that point. In despite of leeching, cold applications to the head, blisters, calomel, and various other remedies, the case terminated fatally, in a week from the time Dr. D. first saw the patient. On dissection, the vessels of the pia mater were rather turgid, but the substance of the brain was little, if at all, softer than natural. There were four ounces of blood in the ventricles. In the pericardium, there was a small quantity of fluid, and the left ventricle of the heart was rather stronger than natural. All the other viscera were healthy.

The above is a phenomenon which is often seen partially—for instance, in the arm, and it proves, beyond a doubt, that the force of the heart may and does act all round the course of the circulation. If the heart be capable of producing a pulse in the veins, under extraordinary circumstances, how can we doubt that it acts in propelling the blood more or less in the veins, under ordinary circumstances. No idea can be more erroneous than that of Bichat and others, that the heart ceases to act after the blood arrives in the capillary system.

### ART. III.

*Case of unusual Constipation.* By JOHN CRAMPTON, M.D.

It is astonishing to observe the difference of constitutions in respect to constipation and relaxation of the bowels. Many are uncomfortable if they have not three or four evacuations daily—while others are never in better health than when they are confined to one or two motions in the week.

The subject of the case now under consideration was a slender, but originally healthy and active young lady, who, after an attack of scarlatina, several years ago, was threatened with phthisis pulmonalis, and became so greatly emaciated, that death seemed inevitable. A removal, however, to the air of MALLOW produced a great amelioration, though the emaciation still continued, together with cough, heavy expectoration, aphthæ in the mouth and fauces, red and sore tongue, occasional diarrhœa. She seldom left her apartment; and, in this way, her health remained stationary for nearly seven years, with every now and then a fresh cold, and an exasperation of her complaint.

In Feb. 1819, she was attacked with a severe diarrhœa, attended with considerable pain, for which she would take no medicine, her own physician being out of the way, and no other being supposed to be acquainted with her constitution. The diarrhœa, however, ceased, and was succeeded by an opposite condition—constipation, attended with pain in the bowels. Peritoneal inflammation, first in a chronic, and afterwards in a subacute form, appeared to have been established, and in this state Dr. C. found his patient when he returned. Her illness had now assumed such a threatening aspect, that he thought every day would be her last. Although no absolute obstruction appeared to exist, yet there was reason to apprehend that "extensive adhesions had taken place between the convolutions of the intestines." She rejected most of the little nourishment she took by the stomach, and emaciation advanced to the last degree. The symptoms then remained stationary for a considerable time, and, circumstances requiring the family to remove 50 miles from Dublin, the patient was conveyed by the canal to her new residence, and bore the journey better than was expected. In the country, the abdomen continued tender and inflated, and she seldom had an evacuation oftener than once a week, all medicines being rejected by the stomach, as was almost the whole of her sustenance. She often brought up fæcal matters by vomiting, and also liquids of a urinous smell and taste, there being very little secretion from the kidneys. Notwithstanding this deplorable condition, it became evident that she bore her sufferings with greater facility—the symptomatic fever subsided—the pulse became natural—she was able to retain sufficient liquid food—she regained some flesh—the vomiting was less severe, but still continued to recur daily; and the intervals between the alvine evacuations became greater. The catamenia were still regular. She has either lost the use of her lower extremities, or is averse to using them. She is now in the 37th year of her age, and has been in the state described for the last seven years. During the last eight months she has had no evacuation from her bowels, and only two or three during the preceding year. She scarcely passes any urine. "There is no possibility that any deceit can be practised, as she sleeps in a room with a confidential attendant, and is watched closely by her parents and her sister." She is irritable and obstinate—makes no effort to amuse herself—yet entertains confident hopes of recovery.

Dr. Crampton met with a case a good deal similar in Steevens' Hospital. The patient, a female, had rarely any motions, and seldom passed water. She lived for several years, often vomiting up urinous fluids. On dissection, the upper part of the colon was found greatly distended, and a stricture in the lower portion of that gut, almost amounting to an obliteration. The bladder was thickened, and otherwise diseased in structure.

### ART. IV.

*Observations on an Affection of the Mouth in Children.* By THOMAS CUMING, M.D. Assistant-Physician to the Institution for the Diseases of Children, &c.

The affection in question is a peculiar kind of ulceration of the gums and cheek, to one variety of which, authors have given the name of cancrum oris. In general, it commences in the gums, and extends to the lips and cheek; but sometimes takes the reverse course. It is most liable to attack during the first dentition; but is frequently met with in children from three to seven years of age.

"When the disease occurs in infants on the breast, it is generally attended with a purplish and spongy appearance of the gums and roof of the mouth, and the ulceration, which lays bare the necks of the teeth, both externally and internally, is of a greenish or ash colour, and very much disposed to bleed. The salivary discharge is increased; the tongue is white; the mouth feels hot; the bowels are for the most part confined, and the child in general labours under a greater or less degree of fever. I have not seen this form of the disease previously to the irruption of the four superior incisors, but I have frequently seen it when the child had only six or eight teeth; and I have constantly observed that when it occurs thus early, it is always the upper gum that is first and principally attacked. This I consider to be the mildest and most manageable form of the disease. As the bowels are for the most part confined, the necessity of a purgative is clearly indicated, and I have generally found that after free alvine evacuation the fever subsides, the mouth becomes cool, the gums lose their red and spongy appearance, and the ulceration speedily heals. In such cases as the above I have seldom found it necessary to make use of any local application. Where, however, the ulcers seem indolent, and little inclined to heal after the repeated administration of purgatives, a little honey of borax, or a mixture of muriatic acid in honey, in the proportion of a drachm to the ounce, may be advantageously applied by means of a feather or camel's hair brush to the ulcerated surface. This disease is very apt to return when the state of the bowels is not particularly attended to. As the biliary secretion seems frequently to be defective either in quantity or quality, I consider small doses of mercurials, followed by occasional aperients, to be amongst the most likely means of confirming the recovery and preventing a relapse." 332.

The most formidable variety is that which occurs in children between twenty months and seven years of age—the subjects being generally of a pale, sallow, or bloated and unhealthy appearance, with irregular bowels, and having had scanty food and bad cloathing. The disease is more observed at the close of the exanthemata than at that of any other acute affection. Dr. Hall, who has published a paper on this subject in the Edinburgh Journal, makes similar observations. In this variety, the ulceration is usually confined to one side of the mouth—is extremely foul—spreads rapidly to the lips and cheek, destroying apparently by gangrene and absorption. If the disease continue long, the teeth fall out, in consequence of the devastation of the gums and alveolar processes. In some cases, the jaw-bone is destroyed. The tongue takes on a similarly diseased action, and is wholly or partially destroyed, so that the unhappy patient exhibits the most horrible spectacle that can be imagined. The disease, however, is frequently in a milder degree than this.

Dr. Hall queries whether this disease may not be caused by the calomel so frequently administered to children in the present day. Dr. Cuming, having observed the cancrum oris in a child after the administration of calomel pretty freely for a hydrocephalic affection, was of a similar opinion with Dr. Hall; but, says he, "I have seen so many cases since of a precisely similar kind, where there was no reason to suppose that mercury had been administered, at least to any extent, that I am strongly disposed to doubt whether there be any necessary connexion between the appearance of the disease and the previous administration of mercury." Indeed our author frequently has recourse to mercury, both as an alterative and a purgative for its removal.

The treatment appears to consist chiefly in clearing the bowels and keeping the secretions in as healthy a state as possible, by the "alternation of mild mercurials with aperients." The local applications which Dr. C. has found most useful are, the black wash, and a dilute solution of muriatic acid in honey—a drachm to the ounce. As soon as the general health is established, the ulceration assumes a healthy appearance and heals. In that variety, however, in which gangrene is predominant, the fatal termination is almost certain, whatever means we may use.

### Art. V.

*A Case of Ruptured Cæcum, which terminated fatally in* 48 *hours after the Accident.* By John Speer, Surgeon to the Essex Convict Hulk.

Mr. Speer was summoned to a strong and muscular man, 35 years of age, whom he found labouring under pain in the abdomen increased by pressure—constant vomiting of a yellowish fluid—cold extremities—no pulse at the wrist—retention of urine—shrunk countenance—bowels constipated for forty-eight hours. He had been wrestling with a neighbour the preceding evening, and, after a severe struggle, threw his antagonist on the ground, and fell with his belly on his adversary's bent knee. The contusion was in the region of the umbilicus. He felt as if something had given way, and immediately fainted. When he recovered from the deliquium, the vomiting and pain in the abdomen appeared. In this state he was conveyed in an open cart the distance of five miles, and no medical aid was procured till the succeeding morning.

Mr. Speer attempted to take some blood from the arm, but faintness occurred before three ouncess were abstracted. The warm bath gave some temporary relief. The pain suddenly ceased in the evening, and he lingered out till the next day, when he expired.

The abdomen being opened, a quantity of the contents of the intestines was found extravasated, through an aperture in the cæcum, which was ruptured. The aperture was two inches in circumference, with ragged edges, and evidently the result of the contusion. It was surrounded with marks of extensive inflammation, and false membranes were thrown out on the peritoneal surfaces.

### ART. VI.

*A Case of Cynanche Laryngea, in which the Operation of Tracheotomy was performed in March,* 1825, *and a Canula worn up to the date of this Report.* By FRANCIS WHITE, one of the Surgeons of St. Mary's Hospital, &c.

Mr. White was called to a man, 33 years of age, labouring under all the symptoms of laryngeal inflammation, on the 10th March, 1825. There was no external swelling—the fauces were redder than natural, and slightly œdematous—no ulceration, no difficulty of deglutition. The breathing was hard and hissing—the cough of a suffocative character—the region of the larynx painful on pressure. He was bled from the arm, had a strong purgative administered, and inhaled the steam of warm water. He was also directed to take two grains of calomel, with some antimonial powder and ipecacuan every four hours. 11th. No better. 12th. Evidently worse. Leeches were applied—a large blister over the sternum—the calomel pills to be continued. 13th. All the symptoms exasperated—he was unable to speak—suffocation imminent —mercury has touched the mouth. All other remedies than the knife were now out of the question, and therefore the operation of tracheotomy (not laryngotomy) was performed at 11 o'clock this morning. When the trachea was slit open, much muco-purulent matter was expelled with the air. Lateral slips of cartilage were then cut away, leaving ample space for the patient to breathe and expectorate. The introduction of a silver canula caused too much irritation, and could not be borne; but the largeness of the aperture rendered it unnecessary. The improvement of the patient's condition was now evident, and a careful pupil was left with him to keep the opening free from accumulations of mucus. A severe ptyalism took place soon after the operation, which harrassed the patient much. In six days from the tracheotomy, the wound began to close, and then a canula was introduced and borne without inconvenience. So much mischief had been occasioned by the original inflammation, that the patient has never been able to breathe through the natural passage—he is consequently compelled to wear an ivory tube, through which he breathes with ease. It is now two years since the operation, and during that period the tube has given no inconvenience, or prevented the man from working at his trade. The perforation is lined with a smooth fine cuticle—the rima glottidis still remains closed to inspiration, though it yields when a slight expiratory force is used.

There are now three or four living with tubes in their throats—including the original patient of this kind, Mr. Price of Portsmouth, who has worn the tube twelve or thirteen years.

### Art. VII.

*Observations respecting an Ulcer of a peculiar Character, which attacks the Eyelids and other Parts of the Face.* By Arthur Jacob, M.D. Surgeon to Sir Patrick Dun's Hospital, &c. &c.

Dr. Jacob observes that the characteristic features of this ulcer are, the extraordinary slowness of its progress—the peculiar condition of its edges and surface—the inconsiderable sufferings produced by it—its incurable nature except by extirpation—and its not contaminating the neighbouring lymphatic glands. In one case of three which our author has observed, the disease had existed for four years, and now presents no remarkable difference from what it exhibited six months ago. The eye-ball, exposed and dissected out by the ulceration, remains precisely in the same state, and the edges occupy the same situation as at that period. In another case the disease has existed for 23 years, without ever having healed. In this case also the eye-ball has been exposed for nearly a year, and has not yet been totally destroyed. In the 3d case, the disease continued nine years prior to the death of the patient from another cause.

There is no lancinating pain, the principal distress appearing to arise from the exposure, by ulceration, of the nerves and other sensible parts. In no case did the disease incapacitate the patients from following their usual occupations. The origin of the disease, in two of the cases, could not be ascertained. In the third case, it commenced in an old cicatrix, the consequence of confluent small-pox, at the inner angle of the eye.

"This disease may be observed under two very different conditions, either in a state of ulceration, or in a fixed state, in which no progress is made toward healing. In this latter condition the parts present the following appearances: the edges are elevated, smooth and glossy, with a serpentine outline; and are occasionally formed into a range of small tubercles or elevations; the skin in the vicinity is not thickened or discoloured. The part within the edges is in some places a perfectly smooth, vascular, secreting surface, having veins of considerable size ramifying over it; which veins occasionally give way, causing slight hæmorrhage; in other places the surface appears covered by florid healthy-looking granulations, firm in texture, and remaining unchanged in size and form for a great length of time. The surface sometimes even heals over in patches, which are hard, smooth, and marked with the venous ramifications to which I have alluded. This healing may take place on any part of the surface, whatever may be the original structure; in the case from which I have had this drawing made, the eye-ball itself, denuded as it is by ulceration, is partially cicatrized over. When the ulceration commences it proceeds slowly, cutting away all parts indiscriminately which may be in the direction in which it spreads; the surface in this state is not so florid, and presents none of the glistening or granulated appearance above noticed; the pain is generally greater at this period. It appears also that there is a tendency to reparation, exclusive of the cicatrization which I have mentioned: there is a deposition of new material, a filling up, in certain places, which gives a uniformity to the surface which should otherwise be very irregular, from the nature of the parts destroyed. When the disease extends to the bones, they sometimes exfoliate in scales of small size, but more generally they are destroyed, as the soft parts, by an ulcerative process. The discharge from the surface is not of the description called by surgeons unhealthy or sanious, but yellow, and of proper consistence; neither is there more fetor than from the healthiest sore, if the parts be kept perfectly clean, and be dressed frequently. There is no fungous growth, nor indeed any elevation, except at the edges, as already noticed, and even this is sometimes very inconsiderable. There is no considerable bleeding from the surface, and when it does occur, it arises from the superficial veins giving way, and not from sloughing or ulceration opening vessels; sometimes the surface assumes a dark gangrenous appearance, which I have found to arise from the effusion of blood beneath. I have not observed that the lymphatic glands were in the slightest degree contaminated, the disease being altogether extended by ulceration from the point from whence it commences." 236.

From the above description, it will be evident that the disease is of a peculiar nature—that it is not genuine carcinoma, nor lupus, nor noli me tangere. It is equally distinct from the cauliflower-like fungous growth which occasionally attacks old cicatrices.

Dr. J. has tried a variety of remedies, internal and external, without the slightest success. It remains, therefore, to be determined by future experience, whether it is remediable by any thing but the knife. Mr. Colles, however, informs our author that he succeeded, in a case which had not extended to the eye-lids, by the application of a powerful escharotic, covering up the eye during the operation of the remedy, with gold-beater's leaf.

### ART. VIII.

*Cases of a fatal Erethism of the Stomach, with Observations.* By JOHN CHEYNE, M.D. Physician-General.

*Case* A. An athletic young man, aged 19 years, who had exposed himself to heats and colds, in July and August, and who had eaten a great quantity of fruit, was taken ill on the 11th of the last-mentioned month. On the 13th, Dr. C. found him with flushed face and white tongue, pulse 90, periodical pains at the umbilicus, thirst, sickness, no tension nor tenderness in any part of the abdomen. He had taken rhubarb and calomel without effect. Fifteen grains of calomel and two of opium were made into six powders, and one ordered to be taken every two hours. A purgative enema. In the evening he was bled to 30

ounces. 14th. Having taken all the pills, he next took salts in tamarind water, which procured large, fluid, and bilious motions. 15th. Slight salivation. 16th, 17th, 18th. Passed bilious stools freely. The abdomen was without pain, tension, or tenderness—stomach still irritable. Saline preparations—blisters—laudanum, &c. 19th. Fever—vomiting of ropy, green, insipid liquor. Bled to 16 ounces; the calomel and opium to be continued. 20th. Pulse softer—stomach less irritable—vesicular eruption round the mouth. 21st. Refused the pills. From this time he went on from bad to worse, notwithstanding leeches, blisters, and many other remedies, and he died with black vomit, there being neither pain, tenderness, nor tension of the abdomen at any time. No dissection.

*Case* B. A young lady became affected with sickness, and vomiting of a green ropy fluid, her health having been previously good. On the 3d day, the tongue was white, the edges clean and florid, throat sore—frequent discharges of green fluid from the stomach. In the evening, there was epigastric tenderness on pressure, and twenty leeches were applied to the abdomen. *4th day.* Excessive bleeding from the leeches—strength sunk—extremities cold—pulse feeble and rapid—overpowering sickness, and vomiting of green fluid. Enema, with laudanum—half a grain of opium every hour, *ad quartam vicem*—fomentations—blister to the abdomen. *5th day.* Has slept some in the night, but the sickness is now uninterrupted. She died in the course of the night. No dissection.

*Case* C. "D—— B——, of a saturnine complexion, sedentary habits, and rather costive bowels, although not confined to the house, and not without appetite, had been affected with occasional sickness and vomiting of every kind of food for fourteen days; the food thrown up was frequently sour.

"Dec. 14th, 1823. I prescribed pills of *Aloes, Rhubarb, and Bicarbonate of Soda.*

"Dec. 15th. In the night he vomited a yellow bitter fluid, I presume bile; in the morning the discharge from his stomach consisted of some undigested chicken broth, which he took on the 14th, and a quantity of green ropy fluid, exactly of the colour of the green baize floor cloth. He said the pills made him sick. *Water in sips; a grape to allay thirst; Enemata; Vesicatorium Epigastrio.*

"December 16th. If he took more than two or three grapes at once, he vomited them. P. 76, soft; no heat of skin; tongue swollen, furred and dry in the centre. Having discovered a tinge of blood in the water with which he had washed his mouth, I was led to examine his throat, which appeared slightly inflamed. Urine turbid; stools produced by the glysters natural, with a proper quantity of healthy bile. *Lemon ice; iced water sparingly; Blister to the left hypochondrium. Twelve leeches to the anus.*

"December 17th. The leeches bled so profusely within the rectum, that when he went to stool he passed from half a pint to a pint of blood which had lodged there. He evinced a dislike to grapes; he took two or three spoonfuls of tea, which he said was too strong, and produced uneasiness of his stomach. In the course of the day he took a glass of lemon ice and the same quantity of pump water, which he preferred to every thing, and with which he was constantly rincing his mouth; he had no complaint but of thirst; two aphthous spots were discovered on the inflamed uvula and velum; he had three liquid, but otherwise natural stools in the night. P. 76; skin temperate. *Various gargles were used; glysters of milk, &c.*

"December 19th. Tongue less swelled; throat much inflamed and beset with aphthous spots; no vomiting since the night of the 16th. He took *a few ounces of human milk and a little ice. Blisters to the angles of the jaw. Glysters, gargles, &c.*

"December 20th. The aphthous spots increasing in number; debility great. *Arrow root with a little sherry. Continue milk and Cinchona glysters.*

"December 21st. Aphthous spots had completely overspread the whole of the palate. Countenance haggard; tarsi inflamed; an eruption of small papulæ on the back and breast.

"December 22d. Pulse 120; weak; strength sunk.

"December 23d. Refuses to take nourishment.

"December 24th. Oppressed breathing.—Death.

"DISSECTION performed on the morning of the 25th.

"The body was emaciated; the abdomen retracted. The gall-bladder was full of dark bile; the stomach, large and flaccid, contained nearly a pint of green fluid. Its mucous membrane presented a peculiar appearance; the veins were unusually turgid; the surface, particularly at the great extremity, was of a dark mahogany colour; the colour was owing to vascular distension, and general extravasation into the submucous tissue. The same appearance of vascular distension and extravasation, but in a lesser degree, was observable in the intestines. The mucous membrane of the œsophagus was of a deep red colour, and highly vascular." 259.

*Case* D. This was more fortunate. A young gentleman, after anxiety of mind, was attacked (30th of May) with pain in the upper part of the abdomen, soon after taking a draught of ale, while on a fatiguing journey. The pain was attended with sickness, and vomiting of green fluid. 31st. An apothecary exhibited a large glyster, which brought away a discharge of hardened fæces, after which, 3 grains of calomel were given. Dr. C. now saw the patient. There was no tension, and but little pain, unless when considerable pressure was used—pulse 80—tongue furred, rather swoln, but moist. Had vomited nearly a quart of amber-coloured fluid, like melted jelly, tart, but not bitter. Rochelle salts, in solution of soda and lemon-juice—blister to the abdomen—lemonade. *June* 1*st*. The symptoms all alleviated, and, by the 3d, he had no complaint.

*Case* E. This case also terminated favourably. The patient was a young servant girl, who came into the Meath Hospital, bent double with pain in the epigastrium. The pain was fixed and burning, and sometimes so violent as to cause her to cry out. There was no tension of the abdominal integuments, but considerable tenderness on pressure of the epigastrium. Whatever she swallowed was instantly thrown up—pulse 100—skin hot—tongue moist and furred. She had been ill three days. No catamenia for six months. Venesectio ad ℥xx.—enema purgans—small doses of sulphate of magnesia in infusion of senna. While bled, she vomited a considerable quantity of green ropy fluid. On the same day, she was again bled to 16 ounces—all medicines omitted—fomentations to the abdomen. The vomiting continuing, she was bled a third time in the evening, and a blister applied to the region of the stomach. She was ordered a grain of calomel every hour. By the next day, having taken nine grains of calomel, the stomach was so much tranquillized, that she was able to retain a dose of castor oil, which produced a stool. The pulse was 120, the face flushed. Bled again, and a grain of calomel given every four hours.

"There can be little hesitation in considering cases A, B, C, D, which are well entitled to the attention of the pathologist, as specimens of one and the same disease, the essence of which was an erethism of the stomach of a very uncommon kind. The subjects of these cases were three brothers and a sister. The family to which they belonged consisted of six children; two, in addition to those whose cases I have related,—one a young lady of eighteen, who died, before I knew the family, of a similar complaint; at least so I infer from the testimony of her mother, who described her last illness as accompanied with vomiting, which was insuperable, of a fluid of the colour of verdigris; some uneasiness in the epigastric region, and aphthæ covering the pharynx.—The other a young gentleman of 20, who after having laboured for some time under cough, purulent expectoration, and a quotidian remittent, which had greatly reduced his strength, at last sunk, in two or three days, under the symptoms which attended the fatal illnesses of his two brothers and two sisters. It is further remarkable, that the father of this family died in a similar manner; he was not under my care, nor have I been able to obtain a particular account of his death, but I learn from one who was present, that, during the last two or three days of his life, he was much distressed with sickness and vomiting of a bright green fluid.

"During my attendance on the individuals whose cases are marked B, C, and D, I experienced great uneasiness of mind, arising from the inefficacy of the means which were adopted in case A, and the dread of a similar result.

"With respect to the treatment, I cannot affirm that Bloodletting was advantageous in case A; indeed the disease appeared to me to pursue its course uninfluenced by the remedial measures. In case B, the patient appeared to sink, in consequence of the effusion of blood, an occurrence by no means unusual, when bleeding after the application of leeches is not timely suppressed. In case C, I perceived not the slightest benefit from bloodletting, upon which I resolved, should I unhappily have to treat another case of the same kind, to abstain from that remedy, either general or local. Nor was calomel and opium, which, in inflammation of the mucous membrane, next to venesection, is the most powerful antiphlogistic we possess, of any efficacy. In case A, salivation was produced without any decided benefit. It will be perceived, moreover, that all the usual means of quieting the stomach, when irritable, were tried in vain." 264.

Lest these cases, and the remarks on them, should lead to inert treatment in genuine gastritis, Dr. C. has added the case E, as illustrative of the decisive measures which are often necessary in the more acute forms of this dangerous disease. In 25 hours, this girl lost upwards of 60 ounces of blood. "This was an example of acute inflammation of the mucous surface of the stomach, which, judging from the tension and tenderness of the epigastrium, had extended to the serous surface, the affection of the mucous surface, however, which was intense, being the main concern." We recommend the following extract to the consideration of those who think that nothing can be done in diseases without a large quantity of medicine.

"In idiopathic inflammation of the internal surface of the stomach, or when that surface becomes inflamed in the progress of dysentery, gout, or other diseases, I have often directed entire abstinence from medicine of every description, and from fluids even of the blandest nature, until the inflammation has been removed by bleeding, blistering, fomentations, &c. In truth the inflamed stomach is often incapable of retaining even a spoonful of cold water; and at first every description of medicine produces an aggravation of sickness, vomiting, and general distress. In such cases I have repeatedly witnessed the good effects of restraining the patient from drinking, and of withholding medicine for one, or even two days. We can then, with advantage, administer calomel in doses of one grain repeated every hour, or of four or five grains with half a grain of the watery extract of opium every third or fourth hour, which we may alternate with a solution of Rochelle salts with soda, to which lemon juice may be added; but before having recourse to these means of resuscitating secretion, we must make sure that we have sufficiently reduced inflammatory excitement. If thirst is urgent, as is often the case in inflammation of the mucous membrane of the stomach,—most painfully urgent it often is, it may be assuaged by one mouthful of water, or by putting a bit of ice in the mouth; if there is much *dry retching*, a drink, consisting of water, lime water and milk, in equal proportions, may be given, which, while it quenches thirst, will sometimes lessen the straining to vomit; if these things fail, we may try a remedy of an opposite kind, namely, weak lemonade, which will often give great relief.

"The same method is applicable to excessive irritability of the stomach in continued fever, when that symptom arises from an inflammatory state of the mucous membrane of that organ, which may take place either with the epigastrium and hypochondria tumid, or without the slightest fullness of the abdomen.* In either case, before we prescribe purgative medicines, let us consider well whether they are likely to produce a *salutary* increase of secretion, or whether it would not be better, before having recourse to these, first to reduce the inflammation of the stomach by the application of leeches, fomentations, blisters, and enemata."

---

* "Very acute inflammation of the mucous membrane of the intestinal canal often exists without tension, or the slightest tenderness of the abdomen; we witness this every day in the dysenteric fever which at present prevails (Sept. 1826). In private practice, I have seen eight fatal cases of dysentery in the course of the last two months, in only one of which was there the slightest tenderness of the abdomen; there was no tension in any of them."

